# Renal Replacement Therapy in the Critical Care Setting

**DOI:** 10.1155/2019/6948710

**Published:** 2019-07-16

**Authors:** Adeel Rafi Ahmed, Ayanfeoluwa Obilana, David Lappin

**Affiliations:** ^1^MB BCh BAO MRCPI MRCP(UK) PGDip (ClinEd), Holder of European Certificate in Nephrology, University Hospital Galway, Galway, Ireland; ^2^MB BCh BAO MRCPI, Specialist Registrar in Nephrology, University Hospital Galway, Galway, Ireland; ^3^MB BCh FRCPI PhD (NUI), Consultant Nephrologist, University Hospital Galway, Galway, Ireland

## Abstract

Renal replacement therapy (RRT) is frequently required to manage critically ill patients with acute kidney injury (AKI). There is limited evidence to support the current practice of RRT in intensive care units (ICUs). Recently published randomized control trials (RCTs) have further questioned our understanding of RRT in critical care. The optimal timing and dosing continues to be debatable; however, current evidence suggests delayed strategy with less intensive dosing when utilising RRT. Various modes of RRT are complementary to each other with no definite benefits to mortality or renal function preservation. Choice of anticoagulation remains regional citrate anticoagulation in continuous renal replacement therapy (CRRT) with lower bleeding risk when compared with heparin. RRT can be used to support resistant cardiac failure, but evolving therapies such as haemoperfusion are currently not recommended in sepsis.

## 1. Introduction

Acute kidney injury (AKI) has a significant association with high mortality in critically ill patients [1]. Acute renal replacement therapy (RRT) provides supportive management for patients with severe AKI and multiorgan failure (MOF). Continuous renal replacement therapy (CRRT), in particular, is utilised for a haemodynamically unstable patient with AKI in an intensive care unit (ICU) setting. This is the standard practice in most ICUs' in the United Kingdom (UK) and the Republic of Ireland [2]. However, limited consensus exists regarding RRT timing, optimum dosing, modality, and therapeutic efficacy beyond AKI management. In light of emerging data, we look to provide an evidence-based review to address these issues.

## 2. Timing of RRT

The timing of initiating RRT in critically ill patients with AKI, in the absence of absolute indications, is challenging [3]. There is a general trend to initiate RRT before a patient develops absolute indications in the ICU setting (Table 1) [4, 5]. This trend is mostly driven by observational data suggesting that relatively earlier initiation of RRT is associated with decreased mortality [6–8]. The primary limitation is the definition of “early” and “delayed” with investigators using a variety of parameters including biochemical markers, like blood urea nitrogen (BUN) and creatinine; clinical markers, like urine output and fluid balance; and the time from onset of developing AKI [9–12].

### 2.1. Observational Data

Data from the Program to Improve Care in Acute Renal Disease (PICARD) trial, a multicentre observational study of AKI, showed that of the patients who required RRT with no previous chronic kidney disease (CKD), initiation at a lower BUN ≤ 76 mg/dL compared to ≥76 mg/dL was associated with lower 14- and 28-day mortality [11, 13]. In another study, investigators used retrospective data to assess a cohort of 130 septic shock patients who required RRT with BUN < 100 mg/dL defined as early RRT and BUN ≥ 100 mg/dL as delayed RRT [14]. The early RRT group had significantly lower mortality at 14, 28, and 365 days compared to delayed RRT.

Observational data examining patients with AKI post cardiac surgery have demonstrated survival benefits with earlier initiation of RRT [10, 12, 15]. Two of the studies used reduced urine output (<100 mL within 8 hours consecutively after surgery, despite furosemide administration) as their criterion for early initiation of CRRT. The attainment of specified BUN, serum creatinine, or potassium thresholds was the trigger for delayed commencement of therapy. The first of these studies examined the outcomes of 64 patients with a high baseline prevalence of class 3 or 4 heart failure and chronic kidney disease (CKD). It reported a survival rate of 78% in the early initiation group, compared with 57% in the delayed initiation group (*P* < 0.05) [10]. The early initiation group was also found to have had a significantly shorter ICU stay (12.5 versus 8.5 days; *P* < 0.05), shorter hospital stay (20.9 versus 15.4 days; *P* < 0.05), and lower rate of multiorgan failure (MOF) (19% versus 29%; *P* < 0.01). The second study, a retrospective analysis of post-CABG AKI using a historical control group, again showed significantly improved survival (77% versus 45%; *P* < 0.016), shorter length of ICU stay (12 versus 8 days; *P* < 0.0001), and shorter length of hospital stay (30 versus 15 days) in the early treatment group (12).

The subgroup analysis of Finnish Acute Kidney Injury study (FINNAKI) compared early RRT with RRT once absolute indication(s) occurred [16]. Initiation of RRT, once an absolute indication had developed (delayed initiation), was associated with higher 90-day mortality compared to early RRT.

Observational data regarding innate renal function recovery based on the timing of initiation of RRT are highly variable with some suggesting no association [17].

### 2.2. Randomized Control Trial (RCTs)

Three RCTs focusing on AKI post cardiopulmonary bypass looked to determine the ideal timing of initiating RRT [18–20]. All the studies were underpowered with a small sample size. In the largest of these with 106 participants, early RRT group was defined as urine output <30 ml/hr for 6 hrs or creatinine clearance <20 ml/min and the delayed RRT group when absolute indications develop. No significant difference in 28-day mortality was noted [20].

An Indian RCT involving 208 patients with primarily tropical infections or obstetric complications-related AKI, compared early RRT group (defined as BUN > 70 mg/dL (>23 mmol/L) or serum creatinine > 7 mg/dL (619 micromoles/l) to delayed RRT (defined as absolute indications) [21]. The trial included a relatively young population with a mean age of 42 years. No significant difference in mortality or renal recovery was noted.

A Canadian study randomized 101 patients with AKI secondary to hypovolemia or sepsis with urine output <6 ml/kg for 12 hours and serum neutrophil gelatinase-associated lipocalin (NGAL) > 400 ng/mL to early or delayed RRT [22]. The early RRT group was initiated within 12 hours of randomization, while the delayed RRT group was initiated when one of the following indications developed: serum potassium >6 mEq/L, serum bicarbonate <10 mEq/L, or PaO_2_/FIO_2_ <200 with evidence of pulmonary oedema. All 48 patients assigned to the early group initiated RRT within 12 hours of randomization. Thirty-three (63%) of 52 patients assigned to the delayed group required RRT, while 25% recovered renal function without the need for any dialysis. There was no significant difference in mortality or renal outcomes between the early and delayed groups.

The Early Versus Late Initiation of Renal Replacement Therapy In Critically Ill Patients With Acute Kidney Injury (ELAIN) trial was a single-centre randomized trial of 231 critically ill patients that tested whether early RRT compared to delayed RRT would reduce all-cause mortality at 90 days [23]. Early RRT was defined as starting RRT within 8 hours of fulfilling KDIGO stage 2 AKI and delayed RRT defined as starting RRT within 12 hours of the development of KDIGO stage 3 AKI or upon an absolute indication developing. Ninety-four percent of the participants were postoperative of which 46% were post cardiac surgery. All patients in the early group received RRT, as did 91% of patients in the delayed RRT group. The median difference in time to RRT initiation from randomization between the 2 interventions was 21 hours. All patients received continuous veno-venous haemodiafiltration (CVVHDF). The primary outcome showed an absolute reduction in 90-day mortality of 15.4% in the early RRT group (39.3%) compared to the delayed RRT (54.7%). Early RRT also led to a higher likelihood of dialysis independence, significantly shorter duration of RRT (9 vs. 25 days), and shortening of hospital stay (51 vs. 82 days). However, the study had a low fragility index of 3, which meant 3 additional deaths in the early group would have made the mortality difference insignificant. A potential for patient selection, inclusion, and treatment bias exists due to its single centre design.

Initiation Strategies for Renal-Replacement Therapy in the Intensive Care Unit (AKIKI) trial was a large multicentre randomized trial of 620 critically ill patients that tested whether delayed RRT compared to early RRT would reduce all-cause 60-day mortality [24]. It involved 31 ICUs from France. Delayed RRT was defined as an absolute indication developing and early RRT as starting RRT within 6 hours of fulfilling KDIGO stage 3 AKI. All the participants were on mechanical ventilation and/or vasopressor support, the majority of whom had septic shock. Ninety-eight percent received RRT in the early group compared to 51% in the delayed group. The median difference in initiating RRT was 57 hrs. The main mode of RRT was intermittent haemodialysis (IHD). The primary outcome showed no significant difference in 60-day mortality between early RRT (48.5%) vs delayed RRT (49.7%). In delayed RRT, the number of RRT-free days was greater (19 vs. 17 days), and the occurrence of catheter-related bloodstream infections was lower (5% vs. 10%), compared with the early strategy. There was no difference in key secondary outcomes including ventilator and vasopressor-free days through day 28, ICU length of stay, hospital length of stay, and dialysis dependence at day 60.

Although AKIKI and ELAIN are the two largest RCTs examining the effect of RRT timing, they were still underpowered to detect significant mortality differences between different RRT initiating strategies. There were some key differences between the two RCTs, which led to the opposing outcomes. The delayed RRT intervention of ELAIN was similar to the early RRT of AKIKI (AKI KDIGO 3). The ELAIN trial primarily had postoperative patients (94%) with AKI, compared to predominantly medical patients with septic shock in AKIKI. The mode of RRT differed as well with CVVHDF used in ELAIN compared to mainly IHD in AKIKI.

The recently published Timing of Renal-Replacement Therapy in Patients with Acute Kidney Injury and Sepsis (IDEAL-ICU) trial, a multicentre RCT involving 488 septic patients, found no significant difference in 90-day mortality between early RRT (<12 hrs of Failure stage of RIFLE criteria, comparative to KDIGO stage 3) and delayed RRT (>48 hrs of Failure stage of RIFLE criteria) [25]. IHD was initially used as a mode of RRT in 45% of patients. Median time to RRT in the early compared to the delayed strategy group was 7.6 hrs and 51.5 hrs, respectively. Patients requiring urgent RRT at time of AKI diagnosis were excluded. Twenty-one percent did not require RRT with delayed RRT strategy due to native renal function recovery; however, a higher rate of severe electrolyte abnormalities was noted. The trial was terminated early by the safety monitoring board on the basis of futility, as around 9500 participants per group would have been required to accurately assess the effect of timing of RRT. The study supports the findings of AKIKI trial (Table 2) [24, 26]. All the RCTs thus far on timing of RRT have a high risk of bias for allocation concealment.

Standard vs. Accelerated Initiation of RRT in Acute Kidney Injury trial (STARRT-AKI) is an ongoing multicentre RCT further evaluating the optimal timing to initiate RRT to have mortality benefits [27].

In conclusion, based on limited evidence, when considering the timing of initiating of RRT in MOF, individual patients physiological reserve based on age, cardiovascular risk factors, pulmonary comorbidities, baseline renal function, and the trend of inflammatory and renal injury markers should be assessed. A delayed strategy of waiting for 48–72 hrs after progressing to AKI KDIGO 3 or until an absolute indication which arises may be applicable to most medical patients with septic shock [24–26]. Patients with low physiological reserve and AKI may benefit from “early RRT” before absolute indications develop, especially fluid overload [11, 28]. There may be potential benefits in initiating RRT before absolute indications develop in AKI associated with severe burns or in postoperative patients, particularly after cardiac surgery [23, 29]. Furosemide stress test (FST) can be used in euvolemic patients with acute tubular necrosis and no underlying CKD, where a bolus of furosemide 1–1.5 mg/kg producing less than 200 ml of urine output over 2 hr reflects an increased risk for progression of AKI and RRT requirement [30, 31]. The Acute Disease Quality Initiative (ADQI) workgroup on CRRT recommended initiating RRT when metabolic and fluid demands exceed total kidney capacity; however, no specific criteria exist to define excessive demand and low capacity (Table 3) [32]. It is important to note none of the major trials assessing septic shock patients have considered KDIGO stage 2 as an indication for early RRT; thus, further studies are required to conclusively establish early RRT has no benefit in this cohort of patients.

## 3. Optimal Dosing of CRRT

The rationale behind very high effluent flow rate (>60 ml/kg/hr) was driven by limited evidence suggesting that removal of inflammatory mediators would improve homeostasis in septic patients. Specific to CRRT in septic AKI, initially small, short-duration RCTs suggested that high effluent flow rate reduced vasopressor requirements, thus improving haemodynamic stability [33–35]. Initial data suggested survival benefit with relatively higher effluent flow rate (>35 ml/kg/hr) [36, 37]. The IVOIRE study consisting of 137 patients with septic shock-associated AKI compared an effluent flow rate of 70 ml/kg/hr with 35 ml/kg/hr. There was no significant difference in vasopressor requirement and 28-day mortality between the two groups [38]. A recent Cochrane review (2017) also concluded that there was no mortality benefit with the use of high-volume haemofiltration (HVHF) compared to standard therapy in AKI secondary to septic shock [39].

However, the discussion is incomplete without mentioning two landmark trials, the VA/NIH Acute Renal Failure Trial Network (ATN) study and the Randomized Evaluation of Normal versus Augmented Level (RENAL) Replacement Therapy Study [40, 41]. These trials compared HVHF versus standard therapy. The ATN study, consisting of 1124 patients, defined high-intensity RRT as IHD or sustained low-efficiency dialysis (SLED) 6 times per week in haemodynamically stable patients, or CVVHDF at an effluent flow rate of 35 mL/kg per hour in haemodynamically unstable patients. Standard intensity treatment was defined as three IHD treatment sessions per week or CVVHDF at 20 mL/kg per hour, respectively. This study found that higher-intensity treatment was not associated with reduced mortality, improved renal recovery, or reduced rate of nonrenal organ failure when compared with less intensive therapy [40]. It also showed significantly more hypotensive episodes requiring vasopressor support and hypophosphatemia in the high-intensity group.

The RENAL study, to date, is the most comprehensive work on the subject of CRRT dosing. 1508 patients were randomly assigned to high-intensity treatment (40 ml/kg/hr) or lower intensity treatment (25 ml/kg/hr). Mode of CRRT was CVVHDF in both groups. Higher intensity of CRRT did not reduce the mortality at 90 days and the rate of dialysis dependence or improve haemodynamic profile but was associated with significantly higher rates of hypophosphatemia [41].

**Figure 1:**
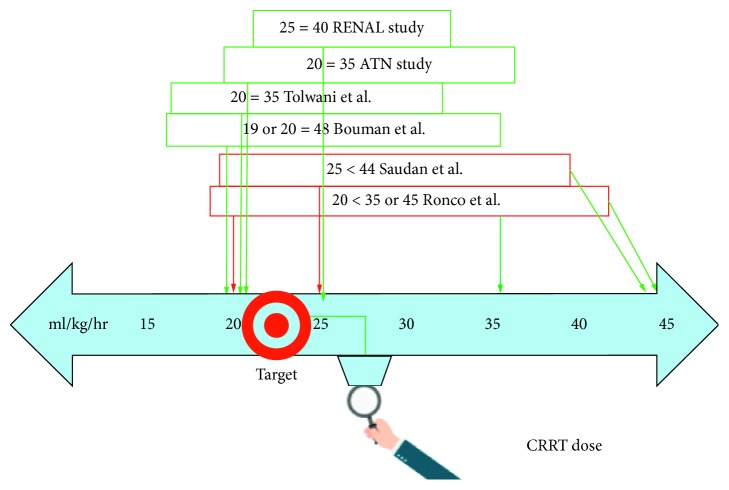
Summary of evidence investigating CRRT dosing(ml/kg/hr). Red box: supporting high effluent flow rate. Green box: supporting lower effluent flow rate.

In conclusion, high effluent flow rates do not affect mortality but are associated with greater electrolyte disturbances, nursing requirements, nutritional demands, and difficulty in maintaining therapeutic drug doses. The current evidence suggests performing CRRT at a minimum delivered dose of effluent flow rate of 20–25 ml/kg/hr (thus prescribing >25 ml/kg/hr to account for treatment interruptions) [40–42]. Further larger trials require regarding optimal CRRT dosing in septic shock patients (Figure 1).

## 4. Modality of RRT

High-quality data directly comparing between CRRT and IHD is lacking due to preferential use of CRRT in haemodynamically unstable patients and lack of CRRT availability in all centres. Current evidence base suggests that CRRT offers no definite benefits to mortality or preservation of renal function when compared to IHD [43, 44].

CRRT, developed in Germany in the 1970s, is generally the preferred choice of RRT in haemodynamically unstable patients due to the potential advantage of more controlled fluid and solute removal, which maintains physiological stability, particularly mean arterial pressure [45–47]. Fluid accumulation is an independent risk factor for poor prognosis in MOF, and CRRT has been shown to be superior at overall fluid removal compared to IHD [13]. Sustained low-efficiency dialysis (SLED) has comparable haemodynamic outcomes and can be considered in MOF [48].

CRRT is associated with a smaller increase in intracranial pressure (ICP) compared to IHD. Thus, it is the preferred option in liver failure awaiting transplantation and acute brain injury, conditions that predispose to cerebral oedema [49–51].

Patients with AKI associated with hypercatabolic states such as severe burns, rhabdomyolysis, posttrauma, or tumour lysis syndrome may benefit from CRRT, as these conditions tend to have more severe haemodynamic instability, metabolic acidosis and fluid overload [29, 52].

IHD is considered the first-line therapy in situations requiring rapid correction of electrolytes (hyperkalaemia), urgent fluid removal, and poisoning.

Overall CRRT, IHD, and SLED are complementary modalities, and their use is based on individual patient condition, availability of nursing staff, equipment, and other resources. Multiple modalities can be used on the same patient. In our practice, we continue to use CRRT in haemodynamically unstable patients in the ICU setting following which we transition them to IHD, once vasopressor support is no longer required (Table 4).

## 5. Extracorporeal Blood Purification beyond AKI in Sepsis

It is widely believed that CRRT removes, or alters the production of, inflammatory mediators and thereby might restore immune homeostasis. Adsorption of inflammatory mediators onto the surface of haemofilters plays a complementary role to simple convection in this process [53, 54]. However, it must be noted that haemofiltration may cause the removal of both proinflammatory and anti-inflammatory cytokines [54]. High cut off (HCO) membranes and high effluent flow rate have been combined with CRRT for cytokine removal in septic patients [55, 56]. Current evidence, however, does not support the routine use of HCO membrane or high effluent flow rate and larger RCTs are required to elucidate any renal or mortality benefits [39]. Haemoperfusion therapy has been considered in septic shock to reduce inflammatory mediators and improve haemodynamic stability. However, the EUPHRATES trial, a multicentre RCT, demonstrated no mortality benefits of using polymyxin B haemoperfusion therapy in septic shock associated with high endotoxin levels. Thus, haemoperfusion in septic shock is currently not recommended [57].

## 6. RRT and Organ Support

In the intensive care setting, AKI occurs in a high percentage of patients with ARDS, cardiogenic shock, and fulminant hepatic failure. Experience using CRRT in the management of these patients has generated interest in whether this intervention can improve outcomes even in patients without AKI, that is, whether CRRT has a supportive role in the management of heart, lung, or liver failure.

### 6.1. RRT in Heart Failure

The UNLOAD trial compared the safety and efficacy of early ultrafiltration (UF) versus standard diuretic therapy in patients presenting with decompensated heart failure (HF). Early UF produced greater fluid and weight loss at 48 hrs with reduced rehospitalization, number of hospital days, and unscheduled clinic visits at 90-day follow-up. Serum creatinine was similar in both groups [58]. The CARRESS-HF trial also looked at safety and efficacy of UF compared to a stepped pharmacological approach based on a targeted urine output (UO) of 3–5 L/day [59]. Intravenous diuretics, thiazide-like diuretics (metolazone), inotropic agents, left ventricular assist device (LVAD), and UF crossover were all used in a stepped-up approach to achieve desired UO. The stepped pharmacological therapy showed superiority to UF in terms of preservation of renal function, lower adverse events, and similar weight loss at 96 hrs. A smaller RCT demonstrated a lower rehospitalization rate at 1 year when UF was used as the primary modality to manage decompensated heart failure [60].

In light of the above results, we broadly agree with the recommendations of American College of Cardiology (ACC) that UF is a reasonable therapeutic modality in refractory heart failure not responding to medical management [61].

### 6.2. RRT in Acute Respiratory Distress Syndrome

Acute respiratory distress syndrome (ARDS) is seen in up to 15% of ICU admission with sepsis being the most common cause [62]. The FACCT trial demonstrated that a conservative fluid management strategy was associated with a reduction in mechanical ventilation and RRT requirement . A post hoc analysis of the same trial showed a decrease in AKI incidence with a conservative fluid strategy [64]. A few small studies have shown reduced ventilatory requirements and improvement in oxygenation with use of CRRT in ARDS [65, 66]. A small study of 53 patients showed the benefit of initiating CRRT within 12 hrs of developing ARDS compared to after 48 hrs [65]. However, in a post hoc analysis of the AKIKI trial, a subgroup of patients with ARDS or sepsis and AKI KDIGO 3 showed no difference in ventilator-free days and 60-day mortality [26]. Early renal recovery was also seen with delayed RRT.

In the presence of positive fluid balance and ARDS, CRRT may be considered to reduce extravascular lung volume and more readily support a conservative fluid management strategy.

### 6.3. RRT in Liver Failure

Application of blood purification strategies to humans with liver failure has mainly occurred in trial settings and is not yet common practice. Experimental approaches have included haemodiabsorption and the molecular adsorbent recirculating system (MARS) [67]. Small studies using these techniques in the management of hepatic failure showed benefit in patients with acute-on-chronic hepatic failure, hepatorenal syndrome (HRS), and even fulminant hepatic failure. In the absence of more robust evidence to confirm these findings, no recommendation can be given to support their routine use in clinical practice [68]. There is no evidence to suggest that RRT increases long-term survival in HRS without liver transplantation. Thus, CRRT should only be utilised if a reversible element to liver failure exists or the patient is listed for liver transplantation [69].

## 7. Anticoagulation in RRT

Extracorporeal circuit clotting is a major issue with CRRT primarily due to the accumulation of proteins in a process called concentration polarization. Significant loss of therapeutic time occurs in replacing clotted haemofilters, which diminishes the efficacy of the treatment. Thus, anticoagulation needs to be individualized for each patient.

Anticoagulation options for CRRT can be divided into four categories.

### 7.1. Anticoagulation Free

Anticoagulation-free RRT should be considered the first line in patients with a high risk of bleeding, such as severe thrombocytopenia, liver dysfunction, coagulation disorder, or any other contraindication to regional anticoagulation (citrate). Haemofilter survival can be prolonged by diminishing haemoconcentration within the filter through predilution replacement fluid, maintaining a filtration fraction percentage of <20% and achieving relatively high blood flow rates (200–300 ml/min) [70, 71]. Patients on therapeutic anticoagulation for another indication (e.g., atrial fibrillation) do not require additional anticoagulation for CRRT.

### 7.2. Regional Anticoagulation

This technique allows prolonged haemofilter life without systemic anticoagulation; thus, it can be utilised in patients with moderate to high risk of bleeding. The available options are regional heparin anticoagulation (RHA) and regional citrate anticoagulation (RCA).

RCA is the preferred modality of anticoagulation in CRRT as recommended in the KDIGO guidelines [42]. Evidence from RCTs suggests lower bleeding rates, reduced cost, and longer haemofilter survival, but similar overall mortality when compared to systemic anticoagulation (heparin-based) [72–74]. Citrate binds to calcium (and magnesium) impairing calcium-dependent procoagulants. Citrate-Calcium complexes are partially removed in the effluent and the rest metabolized in the liver, where they are converted to bicarbonate in 1 : 3 ratio, while calcium is released [75]. Calcium reinfusion via a central line is required to replace lost calcium. Two different preparations of RCA are mainly in use, 4% trisodium citrate or anticoagulant citrate dextrose form A using various protocols. RCA is technically more difficult to perform than systemic anticoagulation with UFH and can lead to hypocalcaemia, hypomagnesaemia, hypernatraemia, metabolic alkalosis (in preserved liver function), and high anion gap metabolic acidosis (in liver dysfunction). It is thus relatively contraindicated in acute liver injury and cardiogenic shock with significant lactic acidosis [75].

RHA involves prefilter infusion of UFH and neutralization with protamine infusion postfilter. RHA is rarely used in practice and carries a higher risk of anaphylactoid reaction, hypotension, leucopenia, thrombocytopenia secondary to protamine, and a shorter haemofilter survival compared to RCA [76].

### 7.3. Systematic Anticoagulation

UFH infusion remains the most widely used anticoagulant, despite being a second-line option (when RCA contraindicated) as per KDIGO guidelines and available evidence suggesting that RCA is superior in haemofilter survival and bleeding risk [42, 74]. Long-term experience, reversibility with protamine, and ease of use maintain its popularity [77]. Heparin-induced thrombocytopenia (HIT) is a complication that can develop within five to ten days of heparin use which requires immediate discontinuation of heparin (discussed further below) [78]. The aim is to maintain activated partial thromboplastin time (aPTT) between 40 and 45 seconds [79].

Low-molecular heparin (LMWH) has the potential advantage of lower bleeding and HIT risk when compared to UFH. However, the experience with its use is limited and requires serial factor Xa monitoring. Limited data from RCTs suggest that LMWH has higher bleeding incidence when compared to RCA, but similar to UFH [80, 81]. Haemofilter survival with LMWH anticoagulation may be slightly superior to UFH [81].

### 7.4. Anticoagulation in Special Situations and Other Agents

Immune-mediated HIT (type 2) is a complication seen with the use of heparin. It is a prothrombotic condition and usually requires systemic anticoagulation on CRRT. Management options include non-heparin-based anticoagulants such as argatroban, lepirudin, and danaparoid [82–86]. Argatroban has hepatic metabolism and may be preferred in AKI. Epoprostenol (prostacyclin) is also a viable option; however, its use in MOF is limited due to a significant risk of hypotension, increasing intracranial pressures and relatively short haemofilter survival [87, 88]. Prostacyclin has a heparin sparing effect and may have a role in centres which utilize UFH as the primary mode of anticoagulation [89].

## 8. Conclusion

The role of RRT in the ICU is primarily to support renal dysfunction with MOF. The strategies to employ it are dynamic and evolving. The optimal time to commence RRT remains a topic of literature debate, surgical patients may benefit from early initiation of therapy, whilst septic patients with MOF may benefit from a delayed strategy. CRRT is considered the first line when managing critically ill patients with haemodynamic instability, although no definitive evidence is available to suggest mortality benefits or renal function preservation when compared to IHD. RRT is a potential therapeutic option when managing resistant congestive heart failure; however, other therapies like haemoperfusion in sepsis have proven ineffective. RCA has comparatively lower bleeding risk compared to heparin-based therapy and remains the choice of anticoagulant in CRRT.

## Figures and Tables

**Table 1:** Conventional indications for renal replacement therapy.

1.1. Fluid overload resistant to diuretic therapy
1.2. Metabolic acidosis (pH < 7.15) refractory to medical management
1.3. Hyperkalaemia (*K* > 6.5 mEq/L) refractory to medical management
1.4. Uraemic symptoms or signs (encephalopathy, pericarditis, and bleeding diathesis)
*Other important indications for RRT*
1.5. Poisoning with a dialyzable drug or toxin
1.6. Hyperthermia refractory to regular cooling techniques
1.7. Life-threatening electrolyte derangements in the setting of acute kidney injury
1.8. Progressive azotaemia or oliguria unresponsive to medical management

**Table 2:** Early versus Delayed RRT strategy: a comparison of ELAIN, AKIKI, and IDEAL-ICU studies.

	ELAIN (23)	AKIKI (24)	IDEAL-ICU (25)
Design	RCT	RCT	RCT
Setting	Single centre	Multicentre (31 ICUs)	Multicentre (29 ICUs)
Population	Predominantly postoperative patients; 47% post cardiac surgery.	Predominantly medical patients with septic shock	Patients with septic shock
(i) Main inclusion criteria	(i) KDIGO stage 2 (ii) NGAL>150 mg/ml (iii) Critical illness including at least one of severe sepsis/vasopressor support/refractory fluid overload/SOFA score >2.	(i) KDIGO stage 3 (Cr>354micromol/L or anuria for >12 hrs or urine output<0.3 ml/kg/hr for 24 hrs) (i) Critical illness (mechanical ventilation or vasopressor)	(i) Failure stage of RIFLE criteria: Oliguria (urine output <0.3 ml per kilogram of body weight per hour for ≥24 hours), Anuria for 12 hours or more, or a serum creatinine level 3 times the baseline level or ≥4 mg per deciliter (≥350 *μ*mol per litre) (ii) Septic shock <48 hrs of commencing vasopressor support
(i) Main exclusion criteria	Preexisting renal disease eGFR <30 ml/min/1.73m2	Preexisting renal disease CrCl < 30 ml/min/1.73m2	End-stage renal disease and obstructive nephropathy
(ii) No. Of patients	231	620	488
*Baseline characteristics*			
(i) SOFA score (early vs delayed)	15.6 vs 16	10.9 vs 10.8	12.2 vs 12.4
(i) APACHE II score (early vs delayed)	30.6 vs 32.7	Not available (NA)	NA
Intervention-early RRT	<8 hrs of AKI KDIGO 2	<6 hrs of AKI KDIGO 3	<12 hrs of failure stage of RIFLE
Control-delayed RRT	<12 hrs of AKI KDIGO 3 or absolute indication	Absolute indications (urea >40 mg/dl, K+>6 mmol/l, pH < 7.15, acute pulmonary oedema, oliguria/anuria >72 hrs)	>48 hrs of failure stage of RIFLE criteria or absolute indications developing
RRT requirement in delayed group (%)	91	51	62
Method of RRT	CVVHDF	Multiple modalities: >50% initially on IHD	Multiple modalities: 45% initially on IHD
*Primary outcome*			
(i) Mortality in early vs delayed RRT	At 90 days: 39.3% vs 54.7%	At 60 days: 48.5% vs 49.7%	At 90 days: 58% vs 54%
(ii) *P* value	0.03	0.79	0.38
*Secondary outcome*			
(i) Duration of RRT early vs delayed (median days)	9 vs 25	NA	4 vs 2
(ii) Ongoing requirement for RRT	At 90 days: 13% vs 15%	At 60 days: 2% vs 5%	At 90 days: 2% vs 3%
Conclusion	Early RRT compared with delayed initiation of RRT reduced mortality over the first 90 days.	No significant difference in mortality between an early and delayed strategy for the initiation of RRT therapy. A delayed strategy averted the need for RRT in a large number of patients.	No significant difference in 90-day mortality between early and strategy of RRT among septic shock patients.

**Table 3:** 17^th^ Acute Disease Quality Initiative (ADQI) consensus on patient selection and timing of CRRT (2016) [32].

*Consensus statement 1.1*: Acute RRT should be considered when metabolic and fluid demands exceed total kidney capacity
*Consensus statement 1.2*: Demand for kidney function is determined by nonrenal comorbidities, the severity of the acute disease, and solute and fluid burden.
*Consensus statement 1.3*: Total kidney function is measured using a variety of different methods. Changes in kidney function and duration of kidney dysfunction can be anticipated by markers of kidney damage.
*Consensus statement 1.4*: The demand-capacity imbalance is dynamic and should be evaluated regularly
*Consensus statement 1.5*: For patients requiring multiple types of organ support, decisions about initiating or withholding RRT should be considered together with other therapies
*Consensus statement 1.6*: Once the decision to initiate RRT has been made, the therapy should be started as soon as possible, typically within less than 3 h.
*Consensus statement 2.1*: Selection of RRT modality depends on the capability/availability of the technology, its inherited risks, and the current needs of the patient
*Consensus statement 2.2*: Continuous types of RRT are recommended in situations where shifts in fluid balance and metabolic fluctuations are poorly tolerated. Intermittent and prolonged intermittent types of RRT have a role in situations where rehabilitation or mobilisation is the priority, and fluid and metabolic fluctuations can be tolerated
*Consensus statement 2.3*: Availability of technologies is determined by local regulations, local resources, including staff and their training/experience and laboratory support, and financial constraints. The choice of technologies that should be made available must balance these issues.
*Consensus statement 3.1*: In situations where other extracorporeal therapies are required, continuous RRT is recommended and integrated systems are preferred over parallel systems.
*Consensus statement 4.1*: Transition of modalities should be considered if the demand-capacity imbalance or treatment priorities have changed and can be met better by an alternative technique.
*Consensus statement 5.1*: RRT should be discontinued if kidney function has recovered sufficiently to reduce the demand-capacity imbalance (current and expected) to acceptable levels or the overall goals of treatment have changed.
*Consensus statement 5.2*: To determine sustained recovery of kidney function, we recommend monitoring of urine output and SCr during RRT.
*Consensus statement 5.3*: For patients requiring multiple types of organ support, decisions about withdrawing RRT should be considered together with other therapies

**Table 4:** Comparison of various RRT modalities.

	IHD	SLED	CRRT
Cost	+	++	++++
Duration	4 hrs daily /alternate days	6–12 hrs daily /alternate days	24 hrs (though achieves 16 hrs on avg.) Continuous
Haemodynamic instability	Least suitable	Good	Most compatible
Compatible with extracorporeal life support	No	No	Yes
In raised intracranial pressure	Increases	Can increase	Usually no change
Anti-coagulation	Can be omitted	Can be omitted	Predilution can be utilised to maintain circuit
Serum concentration of renally cleared drugs	Major fluctuations	Some fluctuation	Least fluctuation
Vascular access	AV fistula or nontunnelled or tunnelled catheter	AV fistula or nontunnelled or tunnelled catheter	Nontunnelled or tunnelled catheter
Compatible with supporting large volume infusions (antibiotics, nutrition, etc.)	No	Would need to be daily and longer sessions	Most compatible
Mobilisation	Most compatible	Could be compatible if done at night/rest time	Not compatible—would need to be discontinued.

IHD : intermittent haemodialysis; SLED : sustained low-efficiency dialysis; CRRT : continuous renal replacement therapy.

## References

[1] Chertow G. M., Burdick E., Honour M., Bonventre J. V., Bates D. W. (2005). Acute kidney injury, mortality, length of stay, and costs in hospitalized patients. *Journal of the American Society of Nephrology*.

[2] Ronco C., Zanella M., Brendolan A. (2001). Management of severe acute renal failure in critically ill patients: an international survey in 345 centres. *Nephrology Dialysis Transplantation*.

[3] Mehta R. L. (2016). Renal-replacement therapy in the critically ill - does timing matter?. *New England Journal of Medicine*.

[4] Bagshaw S. M., Wald R., Barton J. (2012). Clinical factors associated with initiation of renal replacement therapy in critically ill patients with acute kidney injury-a prospective multicenter observational study. *Journal of Critical Care*.

[5] Clark E., Wald R., Adeera L. (2012). Timing the initiation of renal replacement therapy for acute kidney injury in Canadian intensive care units: a multicentre observational study. *Canadian Journal of Anesthesia/Journal Canadien D’anesthésie*.

[6] Seabra V. F., Balk E. M., Liangos O., Sosa M. A., Cendoroglo M., Jaber B. L. (2008). Timing of renal replacement therapy initiation in acute renal failure: a meta-analysis. *American Journal of Kidney Diseases*.

[7] Bagshaw S. M., Uchino S., Bellomo R. (2009). Timing of renal replacement therapy and clinical outcomes in critically ill patients with severe acute kidney injury. *Journal of Critical Care*.

[8] Nascimento G. V. R. D., Balbi A. L., Ponce D., Abrão J. M. G. (2012). Early initiation of dialysis: mortality and renal function recovery in acute kidney injury patient. *Journal Brasileiro de Nefrologia*.

[9] Gettings L. G., Reynolds H. N., Scalea T. (1999). Outcome in post-traumatic acute renal failure when continuous renal replacement therapy is applied early vs. late. *Intensive Care Medicine*.

[10] Elahi M., Lim M. Y., Joseph R. N., Dhannapuneni R. R., Spyt T. J. (2004). Early haemofiltration improves survival in post-cardiotomy patients with acute renal failure. *European Journal of Cardio-Thoracic Surgery*.

[11] Liu K. D., Himmelfarb J., Paganini E. (2006). Timing of initiation of dialysis in critically ill patients with acute kidney injury. *Clinical Journal of the American Society of Nephrology*.

[12] Demirkilic U., Kuralay E., Yenicesu M. (2004). Timing of replacement therapy for acute renal failure after cardiac surgery. *Journal of Cardiac Surgery*.

[13] Bouchard J., Soroko S. B., Chertow G. M. (2009). Fluid accumulation, survival and recovery of kidney function in critically ill patients with acute kidney injury. *Kidney International*.

[14] Carl D. E., Grossman C., Behnke M., Sessler C. N., Gehr T. W. B. (2010). Effect of timing of dialysis on mortality in critically ill, septic patients with acute renal failure. *Haemodialysis International*.

[15] Bent P., Tan H. K., Bellomo R. (2001). Early and intensive continuous haemofiltration for severe renal failure after cardiac surgery. *Annals of Thoracic Surgery*.

[16] Vaara S. T., Reinikainen M., Wald R., Bagshaw S. M., Pettilä V., The FINNAKI Study Group (2014). Timing of RRT based on the presence of conventional indications. *Clinical Journal of the American Society of Nephrology*.

[17] Vats H. S., Dart R. A., Okon T. R., Liang H., Paganini E. P. (2011). Does early initiation of continuous renal replacement therapy affect outcome: experience in a tertiary care center. *Renal Failure*.

[18] Durmaz I., Yagdi T., Calkavur T. (2003). Prophylactic dialysis in patients with renal dysfunction undergoing on-pump coronary artery bypass surgery. *Annals of Thoracic Surgery*.

[19] Sugahara S., Suzuki H. (2004). Early start on continuous hemodialysis therapy improves survival rate in patients with acute renal failure following coronary bypass surgery. *Hemodialysis International*.

[20] Bouman C. S. C., Oudemans-van Straaten H. M., Tijssen J. G. P., Zandstra D. F., Kesecioglu J. (2002). Effects of early high-volume continuous venovenous hemofiltration on survival and recovery of renal function in intensive care patients with acute renal failure: a prospective, randomized trial. *Critical Care Medicine*.

[21] Jamale T. E., Hase N. K., Kulkarni M. (2013). Earlier-start versus usual-start dialysis in patients with community-acquired acute kidney injury: a randomized controlled trial. *American Journal of Kidney Diseases*.

[22] Wald R., Adhikari N. K. J., Smith O. M. (2015). Comparison of standard and accelerated initiation of renal replacement therapy in acute kidney injury. *Kidney International*.

[23] Zarbock A., Kellum J. A., Schmidt C. (2016). Effect of early vs delayed initiation of renal replacement therapy on mortality in critically ill patients with acute kidney injury. *Jama*.

[24] Gaudry S., Hajage D., Schortgen F. (2016). Initiation strategies for renal-replacement therapy in the intensive care unit. *New England Journal of Medicine*.

[25] Barbar S. D., Clere-Jehl R., Bourredjem A. (2018). Timing of renal-replacement therapy in patients with acute kidney injury and sepsis. *New England Journal of Medicine*.

[26] Gaudry S., Hajage D., Schortgen F. (2018). Timing of renal support and outcome of septic shock and acute respiratory distress syndrome. A post hoc analysis of the AKIKI randomized clinical trial. *American Journal of Respiratory and Critical Care Medicine*.

[27] Smith O. M., Wald R., Adhikari N. K. (2013). Standard versus accelerated initiation of renal replacement therapy in acute kidney injury (STARRT-AKI): study protocol for a randomized controlled trial. *Trials*.

[28] Libório A. B., Leite T. T., Neves F. M. D. O., Teles F., Bezerra C. T. D. M. (2015). AKI complications in critically ill patients: association with mortality rates and RRT. *Clinical Journal of the American Society of Nephrology*.

[29] Chung K. K., Lundy J. B., Matson J. R. (2009). Continuous venovenous hemofiltration in severely burned patients with acute kidney injury: a cohort study. *Critical Care*.

[30] Lumlertgul N., Peerapornratana S., Trakarnvanich T. (2018). Early versus standard initiation of renal replacement therapy in furosemide stress test non-responsive acute kidney injury patients (the FST trial). *Critical Care (London, England)*.

[31] Chawla L. S., Davison D. L., Brasha-Mitchell E. (2013). Development and standardization of a furosemide stress test to predict the severity of acute kidney injury. *Critical Care*.

[32] Ostermann M., Joannidis M., Pani A. (2016). Patient selection and timing of continuous renal replacement therapy. *Blood Purification*.

[33] Cole L., Bellomo R., Journois D., Davenport P., Baldwin I., Tipping P. (2001). High-volume haemofiltration in human septic shock. *Intensive Care Medicine*.

[34] Ghani R. A., Zainudin S., Ctkong N. (2006). Serum IL-6 and IL-1-ra with sequential organ failure assessment scores in septic patients receiving high-volume haemofiltration and continuous venovenous haemofiltration. *Nephrology*.

[35] Boussekey N., Chiche A., Faure K. (2008). A pilot randomized study comparing high and low volume hemofiltration on vasopressor use in septic shock. *Intensive Care Medicine*.

[36] Ronco C., Bellomo R., Homel P. (2000). Effects of different doses in continuous veno-venous haemofiltration on outcomes of acute renal failure: a prospective randomised trial. *The Lancet*.

[37] Saudan P., Niederberger M., De Seigneux S. (2006). Adding a dialysis dose to continuous hemofiltration increases survival in patients with acute renal failure. *Kidney International*.

[38] Joannes-Boyau O., Honoré P. M., Perez P. (2013). High-volume versus standard-volume haemofiltration for septic shock patients with acute kidney injury (IVOIRE study): a multicentre randomized controlled trial. *Intensive Care Medicine*.

[39] Borthwick E. M. J., Hill C. J., Rabindranath K. S., Maxwell A. P., McAuley D. F., Blackwood B. (2017). High-volume haemofiltration for sepsis in adults. *Cochrane Database of Systematic Reviews*.

[40] Palevsky P. M., Palevsky P. M., Zhang J. H (2008). Intensity of renal support in critically ill patients with acute kidney injury. *New England Journal of Medicine*.

[41] Bellomo R., Bellomo R., Cass A (2009). Intensity of continuous renal-replacement therapy in critically ill patients. *New England Journal of Medicine*.

[42] Khwaja A. (2012). KDIGO clinical practice guidelines for acute kidney injury. *Nephron*.

[43] Nash D. M., Przech S., Wald R., O’Reilly D. (2017). Systematic review and meta-analysis of renal replacement therapy modalities for acute kidney injury in the intensive care unit. *Journal of Critical Care*.

[44] Rabindranath K., Adams J., Macleod A. M., Muirhead N. (2007). Intermittent versus continuous renal replacement therapy for acute renal failure in adults. *Cochrane Database of Systematic Reviews*.

[45] Bagshaw S. M., Berthiaume L. R., Delaney A., Bellomo R. (2008). Continuous versus intermittent renal replacement therapy for critically ill patients with acute kidney injury: a meta-analysis. *Critical Care Medicine*.

[46] Clark W. R., Mueller B. A., Alaka K. J., Macias W. L. (1994). A comparison of metabolic control by continuous and intermittent therapies in acute renal failure. *Journal of the American Society of Nephrology: JASN*.

[47] Kramer P., Wigger W., Rieger J., Matthaei D., Scheler F. (1977). Arteriovenous haemofiltration: a new and simple method for treatment of over-hydrated patients resistant to diuretics. *Klinische Wochenschrift*.

[48] Kovacs B., Sullivan K. J., Hiremath S., Patel R. V. (2017). Effect of sustained low efficient dialysisversuscontinuous renal replacement therapy on renal recovery after acute kidney injury in the intensive care unit: a systematic review and meta-analysis. *Nephrology*.

[49] Davenport A., Will E. J., Davison A. M. (1993). Effect of renal replacement therapy on patients with combined acute renal and fulminant hepatic failure. *Kidney international. Supplement*.

[50] Davenport A., Will E. J., Davidson A. M. (1993). Improved cardiovascular stability during continuous modes of renal replacement therapy in critically ill patients with acute hepatic and renal failure. *Critical Care Medicine*.

[51] Davenport A. (2001). Renal replacement therapy in the patient with acute brain injury. *American Journal of Kidney Diseases*.

[52] Brochard L., Abroug F., Brenner M. (2010). An official ATS/ERS/ESICM/SCCM/SRLF statement: prevention and management of acute renal failure in the ICU patient. *American Journal of Respiratory and Critical Care Medicine*.

[53] Kellum J. A., Song M., Venkataraman R. (2004). Hemoadsorption removes tumor necrosis factor, interleukin-6, and interleukin-10, reduces nuclear factor-*κ*B DNA binding, and improves short-term survival in lethal endotoxemia. *Critical Care Medicine*.

[54] De Vriese A. S., Colardyn F. A., Philippé J. J., Vanholder R. C., De Sutter J. H., Lameire N. H. (1999). Cytokine removal during continuous hemofiltration in septic patients. *Journal of the American Society of Nephrology: JASN*.

[55] Villa G., Chelazzi C., Morettini E. (2017). Organ dysfunction during continuous veno-venous high cut-off hemodialysis in patients with septic acute kidney injury: a prospective observational study. *PLoS One*.

[56] Villa G., Zaragoza J. J., Sharma A., Neri M., De Gaudio A. R., Ronco C. (2014). Cytokine removal with high cut-off membrane: review of literature. *Blood Purification*.

[57] Dellinger R. P., Bagshaw S. M., Antonelli M. (2018). Effect of targeted polymyxin B hemoperfusion on 28-day mortality in patients with septic shock and elevated endotoxin level. *JAMA*.

[58] Costanzo M. R., Guglin M. E., Saltzberg M. T. (2007). Ultrafiltration versus intravenous diuretics for patients hospitalized for acute decompensated heart failure. *Journal of the American College of Cardiology*.

[59] Bart B. A., Goldsmith S. R., Lee K. L. (2012). Ultrafiltration in decompensated heart failure with cardiorenal syndrome. *New England Journal of Medicine*.

[60] Marenzi G., Muratori M., Cosentino E. R. (2014). Continuous ultrafiltration for congestive heart failure: the CUORE trial. *Journal of Cardiac Failure*.

[61] Felker G. M., Mentz R. J. (2012). Diuretics and ultrafiltration in acute decompensated heart failure. *Journal of the American College of Cardiology*.

[62] Estenssoro E., Dubin A., Laffaire E. (2002). Incidence, clinical course, and outcome in 217 patients with acute respiratory distress syndrome. *Critical Care Medicine*.

[63] Wiedemann H. P., Wheeler A. P., Bernard G. R., Thompson B. T., Hayden D., deBoisblanc B. (2006). Comparison of two fluid-management strategies in acute lung injury. *New England Journal of Medicine*.

[64] Liu K. D., Thompson B. T., Ancukiewicz M. (2011). Acute kidney injury in patients with acute lung injury: impact of fluid accumulation on classification of acute kidney injury and associated outcomes. *Critical Care Medicine*.

[65] Han F., Sun R., Ni Y. (2015). Early initiation of continuous renal replacement therapy improves clinical outcomes in patients with acute respiratory distress syndrome. *American Journal of the Medical Sciences*.

[66] Garzia F., Todor R., Scalea T. (1991). Continuous arteriovenous hemofiltration countercurrent dialysis (CAVH-D) in acute respiratory failure (ARDS). *Journal of Trauma: Injury, Infection, and Critical Care*.

[67] Mitzner S., Stange J., Klammt S. (2000). Improvement of hepatorenal syndrome with extracorporeal albumin dialysis mars: results of a prospective, randomized, controlled clinical trial. *Liver Transplantation*.

[68] Ash S. R. (2001). Powdered sorbent liver dialysis and pheresis in treatment of hepatic failure. *Therapeutic Apheresis and Dialysis*.

[69] Allegretti A. S., Parada X. V., Eneanya N. D. (2018). Prognosis of patients with cirrhosis and AKI who initiate RRT. *Clinical Journal of the American Society of Nephrology*.

[70] Baldwin I., Bellomo R., Koch B. (2004). Blood flow reductions during continuous renal replacement therapy and circuit life. *Intensive Care Medicine*.

[71] Joannidis M., Oudemans-van Straaten H. M. (2007). Clinical review: patency of the circuit in continuous renal replacement therapy. *Critical Care*.

[72] Kutsogiannis D. J., Gibney R. T. N., Stollery D., Gao J. (2005). Regional citrate versus systemic heparin anticoagulation for continuous renal replacement in critically ill patients. *Kidney International*.

[73] Zhang Z., Hongying N. (2012). Efficacy and safety of regional citrate anticoagulation in critically ill patients undergoing continuous renal replacement therapy. *Intensive Care Medicine*.

[74] Stucker F., Ponte B., Tataw J. (2015). Efficacy and safety of citrate-based anticoagulation compared to heparin in patients with acute kidney injury requiring continuous renal replacement therapy: a randomized controlled trial. *Critical Care*.

[75] Morabito S., Pistolesi V., Tritapepe L., Fiaccadori E. (2014). Regional citrate anticoagulation for RRTs in critically ill patients with AKI. *Clinical Journal of the American Society of Nephrology*.

[76] Gattas D. J., Rajbhandari D., Bradford C., Buhr H., Lo S., Bellomo R. (2015). A randomized controlled trial of regional citrate versus regional heparin anticoagulation for continuous renal replacement therapy in critically ill adults. *Critical Care Medicine*.

[77] Uchino S., Bellomo R., Morimatsu H. (2007). Continuous renal replacement therapy: a worldwide practice survey. *Intensive Care Medicine*.

[78] Karakala N., Tolwani A. (2016). We use heparin as the anticoagulant for CRRT. *Seminars in Dialysis*.

[79] van de Wetering J., Westendorp R. G., van der Hoeven J. G., Stolk B., Feuth J. D., Chang P. C. (1996). Heparin use in continuous renal replacement procedures: the struggle between filter coagulation and patient hemorrhage. *Journal of the American Society of Nephrology*.

[80] Oudemans-van Straaten H. M., Bosman R. J., Koopmans M. (2009). Citrate anticoagulation for continuous venovenous hemofiltration. *Critical Care Medicine*.

[81] Joannidis M., Kountchev J., Rauchenzauner M. (2007). Enoxaparin vs. unfractionated heparin for anticoagulation during continuous veno-venous hemofiltration: a randomized controlled crossover study. *Intensive Care Medicine*.

[82] Gajra A., Vajpayee N., Smith A., Poiesz B. J., Narsipur S. (2007). Lepirudin for anticoagulation in patients with heparin-induced thrombocytopenia treated with continuous renal replacement therapy. *American Journal of Hematology*.

[83] Link A., Girndt M., Selejan S., Mathes A., Böhm M., Rensing H. (2009). Argatroban for anticoagulation in continuous renal replacement therapy. *Critical Care Medicine*.

[84] Klingele M., Bomberg H., Lerner-Gräber A. (2014). Use of argatroban: experiences in continuous renal replacement therapy in critically ill patients after cardiac surgery. *Journal of Thoracic and Cardiovascular Surgery*.

[85] Lindhoff-Last E., Betz C., Bauersachs R. (2001). Use of a low-molecular-weight heparinoid (danaparoid sodium) for continuous renal replacement therapy in intensive care patients. *Clinical and Applied Thrombosis/Hemostasis*.

[86] de Pont A.-C. J., Hofstra J.-J. H., Pik D. R., Meijers J. C., Schultz M. J. (2007). Pharmacokinetics and pharmacodynamics of danaparoid during continuous venovenous hemofiltration: a pilot study. *Critical Care*.

[87] Fiaccadori E., Maggiore U., Rotelli C. (2002). Continuous haemofiltration in acute renal failure with prostacyclin as the sole anti-haemostatic agent. *Intensive Care Medicine*.

[88] Davenport A., Will E. J., Davison A. M. (1991). The effect of prostacyclin on intracranial pressure in patients with acute hepatic and renal failure. *Clinical Nephrology*.

[89] Kozek-Langenecker S. A., Spiss C. K., Gamsjager T., Domenig C., Zimpfer M. (2002). Anticoagulation with prostaglandins and unfractionated heparin during continuous venovenous haemofiltration: a randomized controlled trial. *Wiener Klinische Wochenschrift*.

